# Prevalence and Pattern of Musculoskeletal Injuries Among Malaysian Hockey League Players

**DOI:** 10.5704/MOJ.2103.004

**Published:** 2021-03

**Authors:** H Manaf, M Justine, N Hassan

**Affiliations:** 1 Centre of Physiotherapy Studies, Universiti Teknologi MARA, Puncak Alam, Malaysia; 2 Department of Physiotherapy, Institut Sukan Negara, Kuala Lumpur, Malaysia

**Keywords:** hockey, Malaysia, musculoskeletal injuries, sprain, strain

## Abstract

**Introduction:**

Hockey is a team sport that involves running, sprinting, and making sudden changes in directions of movement to control a ball against the opposing team. Therefore, due to its nature of fast movement, hockey players may be at risk of various musculoskeletal injuries. This study aimed to identify the prevalence and pattern of musculoskeletal injuries sustained among Malaysian Hockey League players.

**Materials and Method:**

Data were collected from 84 field hockey players that participated in the Malaysian Hockey League competition from June 2016 until December 2016. All injuries were recorded by the participating medical team using a structured questionnaire. A descriptive statistical analysis and Chi-Square test were used to explore the prevalence of the injury.

**Result:**

More than half of the players were reported to have lower limb injuries (51.6%). Sprain and strain were the most prevalent injuries (63%) and mostly affected the ankle (29%). Male players sustained more injuries (50.8%) compared to female players (49.2%).

**Conclusion:**

This study suggests that a guideline is needed for injury prevention strategies that will benefit the hockey players in preventing injuries.

## Introduction

Field hockey is one of the world's most competitive and fastest team sports games. Field hockey is a contact sport that is played on artificial turf and natural grass. It involves techniques such as hitting, pushing, flicking the ball, and sudden and frequent changes in direction. Changes made to the rules of field hockey such as the self-pass and high balls have made the game even faster, which in turn may increase the risk of musculoskeletal injuries among players^1^.

A previous study has reported that the number of injuries per 1000 player match hours ranged from 20.8 to 90.9 in men and 23.4 to 44.2 in women^1^. Furthermore, most of the injuries that occurred were to the lower limb. The same study reported that the location of injuries among male hockey players includes thigh and knee (28%), head and face (27%), hand (16%), and ankle (13%). Another study reported that more than 40% of all game injuries include ankle sprains, meniscal tears, tibiofemoral ligament sprains, tibia and fibula fractures, and quadriceps and hamstring strains^2^. This indicates that the most common injury among hockey players is in the legs, thus preventive measures to this location should be given due attention.

Data from Junge *et al,* indicates that males have a higher rate of injury and sustained severe injuries compared to females^3^. Meanwhile, previous studies have reported that the predisposing mechanisms of injures are physical trauma (collision with the ball, stick, and other hockey players) and frequent rotational movements^1,4,5^. In terms of the playing position, goalkeepers had the highest rate of injury followed by the midfielder, forward, and defender^6^. In contrast, one study reported that midfielders had the highest percentage of injuries (27.6%), followed by defenders (23.6%), forwards (22.4%), and goalkeepers (19.5%)^2^. These inconsistent results indicate that more research is needed to explain why these differences exist especially among Malaysian players.

Apart from affecting playing performance, musculoskeletal injuries among field hockey players also incur societal costs and psychological effects^7^. Fear of movement (kinesiophobia) as a result of previous trauma could become an emotional disturbance as it lowers the level of confidence of athletes while in the game. Despite that field hockey is among the most popular games in Malaysia, there is a lack of information on data related to injury rates and types among Malaysian hockey league players. In addition, there is a lack of information on the differences in injury rates among genders and positions. Therefore, the purpose of this study was to determine the prevalence of musculoskeletal injuries in comparisons with gender and playing positions among Malaysian field hockey players. Identification of the prevalence and pattern of injuries among hockey players may provide supportive evidence for developing a guideline for injury prevention strategies that can be referred by players, coaches, strength conditioning specialists, and physiotherapists when dealing with players, especially those at risk for injuries.

## Material and Method

A total of 84 (43 male, 42 female) field hockey players participated in this cross-sectional study. They were recruited using purposive sampling. The inclusion criteria for participants were as follows: (1) players currently participating in a national field hockey league, (2) aged over 18 years, (3) selected as team players for two months prior to the competition and (4) involved ≥2 hours training and played one match per week on natural grass or artificial turf. They were excluded if they did not participate or register in the Malaysian league from June 2016 until December 2016. All players read and signed the informed consent documents before participating in this study. The study protocol was approved by the Research Ethics Committee of Universiti Teknologi MARA (600-IRMI: 5/1/6).

Data collection was conducted at the National Hockey Stadium, Bukit Jalil as it was the main venue for the Malaysian Hockey League in 2016. All participating players were strongly advised to wear minimal protective equipment such as mouthguards, shin, and ankle guards. All injuries were recorded by the participating medical team (doctor or physiotherapist) using a structured questionnaire^8^. Each participating team had their own medical team as this is one of the tournament regulations. The questionnaire consisted of three sections. First, the demographic section collected data such as age, gender, the name of the team; as well as the players’ position (goalkeeper, defender, midfielder, and forward). The second section assessed the injury data including the day of injury; place of injury (indoors, outdoors); phase of play; injury mechanism; localization of injury (upper extremity, lower extremity, and trunk) and type of injury. Next section gathered information about the situation resulting in an injury; the judgments of the referee; diagnosis; treatment (conservative or operative); the use of taping, sick leave; absence time from practice or game; the numbers of physiotherapy sessions and the practice and playing hours per month.

Before data collection, a briefing on how to fill up the questionnaire was completed by the investigator to the medical team involved in the study. The medical team was requested to indicate only musculoskeletal injuries that were related to hockey and not injuries contracted from other sports or recreational activities. An injury was defined as a new musculoskeletal symptom incurred during a practice or game that required team doctors to do a physical examination and prevented the player from returning to the same practice or game, or forced the player to miss a subsequent practice or game^9^. The injured player was examined by the medical team immediately after the incident. The extend of the injury was supported with diagnostic modalities such as Medical Resonance Imaging (MRI) or Computed Tomography Scan (CT-Scan) when required.

IBM SPSS statistical software version 21 was used to analyse the data. A simple descriptive statistical analysis and Chi-Square test was used to explore the frequencies, percentages of demographic data and prevalence of the injury. Cross tabulations were used to compare the players’ positions related to injury as well as gender comparison. The demographic data were recorded in numerical codes. The players’ positions were assigned as 1 “forward,” 2 “midfielder,” 3 “defender” and 4 “goalkeeper.” The injury mechanism option was from 1 to 6 (collision, tackling, falling, overuse, slipping, and others). The localization of injury was recorded in numerical, where it was coded up to 13 (head, neck, face, shoulder, elbow, wrist, finger, hip, knee, ankle, toe, upper back, and lower back).

## Results

**Fig. 1:**
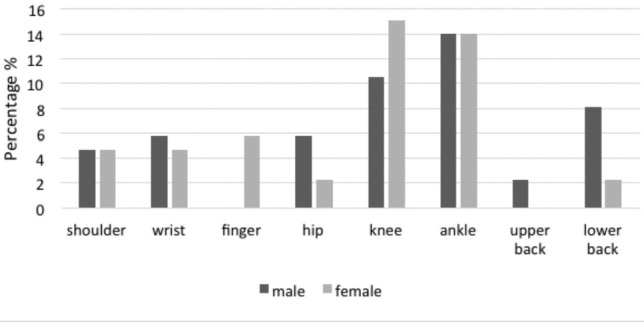
Location of injury based on gender.

Table 1 shows the demographic data of the hockey players. Table II reports the prevalence of musculoskeletal injuries based on location of injury and player’s position. Meanwhile, Fig. 1 shows the location of injury related to gender. Females showed higher injury rates in the knee (15%) while male players sustained injury mostly in the ankle (14%). Consequently, both genders showed a similar result of shoulder injury with 4.7%. In male players, they sustained a higher rate of the upper back and lower back injuries with a percentage of 2.3% and 8.1%, respectively. Another anatomical part of the injury was the hip in which male players showed a higher rate of hip injury (5.8%) compared to females (2.3%).

**Table I:** Demographic data of the hockey players

	Frequency (n)	Percentage (%)
**Gender**		
Female	41	48.8
Male	43	51.2
**Injury Time**		
Game	59	70.2
Training	25	29.8
**Injury Time During Game**		
First half	33	39.3
Second half	51	60.7
**Treatment**		
Conservative	74	88.1
Operative	10	11.9

**Table II:** Prevalence of musculoskeletal injuries based on location of injury and Players' position

Location of Injury	Players' Position				Sum		X^2^
	Forward	Midfielder	Defender	Goalkeeper	N	%	p-value
Ankle	9	8	6	1	24	29	.09
Knee	6	7	6	3	22	26	.65
Lower back pain	4	2	1	2	9	11	.55
Wrist	6	0	2	1	9	11	.03*
Hip	1	3	2	1	7	8	.67
Shoulder	3	3	2	0	8	9	.40
Finger	1	1	3	0	5	6	.28
Total Injuries	30	24	22	8	84	100	

* p≤0.05 was considered to be statistically significant.

Table III shows the prevalence of musculoskeletal injury based on the mechanism of injury across the player’s position. Overall, 38% of the injury mechanism were due to overuse injuries (p =.03) and collisions (16%, p = .04). Defender positions sustained most injuries (9%) from overuse injury mechanisms. A player who was in the forward position mostly had an injury caused by a collision with 9.3% incidence. Other mechanisms of injuries included falling (13%) and slipping (11%).

**Table III:** Prevalence of musculoskeletal injuries based on mechanism of injury and players' position

Mechanism of Injury	Players' Position				Sum		p-value
	Forward	Midfielder	Defender	Goalkeeper	N	%	X^2^
Overuse	4	11	12	3	30	38	.03*
Tackling	6	4	8	1	19	22	.13
Collision	8	3	2	1	14	16	.04*
Falling	5	2	2	2	11	13	.48
Slipping	3	4	1	2	10	11	.57
Total Injuries	26	24	25	9	84	100	

* p≤0.05 was considered to be statistically significant.

**Fig. 2:**
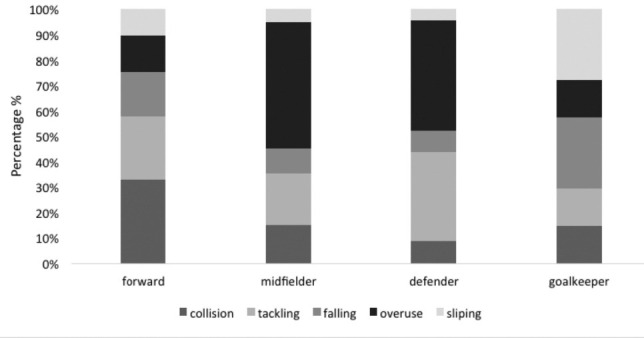
Mechanism of injury according to the player’s position.

Fig. 2 demonstrates the mechanism of injury based on the players’ position. Mechanism of injury based on position showed that the midfielders and defenders were prone to get an injury due to overuse with 11.6% and 9% incidence, respectively. The forward position had their most frequent collision with a 9.3% incidence. Defender, however, predominantly sustained injuries due to tackling with 9.3% incidence. In forward position, 7% of injury mechanisms came from tackling, followed by overuse (5%) and slipping (4%). The goalkeeper was found to have slipping injuries with 2% incidence.

The most prevalent types of injury were sprain and strain across the player’s position (p = .03) Table IV. Sprain and strain were the most common injuries with those in the forward position (23.3%), followed by the midfielder (18.6%), defender (14%), and goalkeeper (6%). Nevertheless, the highest prevalence of overuse injury was shown among the defenders (7%), followed by the forward position (4%), and midfielder (2.3%). The third most common type of injury was the ligament injury, which resulted in 4% incidence among midfielders, followed by a fracture, which occurred mostly among defenders (3%). The least was the meniscus injuries with 2% incidence among defenders and muscle rupture with 2% incidence among midfielders.

**Table IV:** Prevalence of musculoskeletal injuries based on type of injury and players' position

Location of Injury	Players' Position				Sum		p-value
	Forward	Midfielder	Defender	Goalkeeper	N	%	X^2^
Strain and sprain	20	16	12	5	53	63	.03*
Overused	3	2	6	0	11	13	.08
Ligament ruptured	2	3	0	2	7	8	.44
Fracture	2	1	3	0	6	7	.34
Meniscus ruptured	1	1	2	0	4	5	.57
Muscle ruptured	2	1	0	0	3	4	.30
Total Injuries	30	24	23	7	84	100	

* p≤0.05 was considered to be statistically significant.

**Fig. 3:**
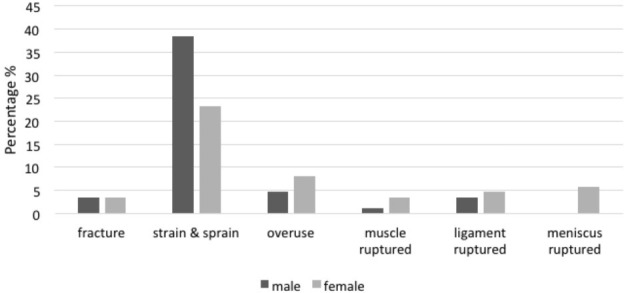
Types of injury based on gender.

Fig. 3 shows the type of injury related to gender. Both genders showed that strain and sprain were the commonest types of injury in female and male. However, both injuries predominantly occurred among male players with 38.4% compared to female players with 23.3%. Results also reported a meniscus injury among female players (5.8%) but none in male players. In terms of mechanism of injuries among genders, overused injuries were the most common occurrence in both genders with 17.4% in males and 11.6% in females. Apart from that, results also showed about 11.6% tackling injuries occurred in men and 10.5% in females, followed closely by collision with 9.3% in males and 7% in females. In addition, female players were also reported to sustain the highest incidence of falling with 9.4% incidence compared to male 3.5%. The least mechanism of injury, which was slipping, was reported to occur about 7% in male players and 4.7% in female players.

## Discussion

To the best of our knowledge, this is the first study to identify the injuries sustained by the Malaysian field hockey players. We found the lower extremities to be the most affected body region (51.6%), of which the predominant site was the ankle (29%). The complexity of the ankle joint structure and the multidirectional forces and movement imposed on ankle joint during sports games could explain why ankle was the most vulnerable site of injury^10,11^. Following the ankle, knee showed to be the second-highest prevalence of injury. The finding, however, is inconsistent with a previous study in which they reported that knee was the most common body site to be exposed to injury rather than the ankle^12^. A previous study found that a rapid change in movement, previous trauma to the knee, shoe, and artificial surfaces could lead to a knee injury during a field hockey game^12-14^.

Based on the current study, sprain and strain were found to be the most prevalent types of injury among field hockey players (63%), with the most occurrence in the ankle (18.6%). This could be due to the high repetitive impact loads that lead to an increase in ligament laxity in the ankle. This current study also suggests that the mechanism of the injury which was overuse (38%) may lead to an ankle injury. Overuse could be related to endurance activity and repetitive performance during field hockey that lead to chronic pain and worsening after completion of the tournament^15^. In addition, training more than 16 hours per week could also contribute to overuse injury^16^.

Regarding the gender, male players were reported to have a higher prevalence of injury compared to female players. The result is consistent with Theilen *et al*^1^ and Murtaugh *et al*^7^ which could be caused by the intensity of the game with more males involved in the sports rather than female. Meanwhile, female players were most prone to get overuse injury^6^. This could be related to lower muscle mass and hormonal changes among female players that made them susceptible to overuse injuries^5^.

Based on playing positions among field hockey players, the current finding revealed that forward position presented with the highest prevalence of injury. However, this finding is inconstant with a study by Murtaugh (2001)^6^, in which the authors found the goalkeeper to have the highest prevalence of injury. He suggested that the high speed of collision from a forward position with a static position by the goalkeeper could be the reason why injuries among goalkeepers were more severe than other positions. Meanwhile, we found that midfielders were more prone to injury as their duration of playing in the field was longer than other players.

The mechanism of injury according to playing positions also varied among each position. The forward position, needing high speed in attempting to shoot a goal; while the defender and the goalkeeper from the opponent team, trying to stop at the same time would subsequently cause a collision and lead to various types of injury^7^. A goalkeeper may also sustain lower limb injury due to slipping while trying to clear the ball from getting into the goal. Midfielders, however, were reported to be prone to get an overuse injury to the upper and lower limb joints as a result of repetitive running and hitting the ball^5^.

The current study presents a few limitations. Firstly, players were recruited among both senior and junior players. Injuries obtained by a junior player may be different from the senior players who have been playing for longer seasons. Secondly, the questionnaire was not equally distributed to all positions. Equal results from all positions might give a different interpretation of injury among field hockey players in Malaysia. This study also did not focus on training hours, types of shoes, as well as types of artificial turf that may provoke risks of injuries.

## Conclusions

The prevalence of injury was noticeably high among field hockey players in Malaysia. The pattern of injury was almost similar to previous reports of field hockey injuries in the literature. This study indicates that Malaysian athletes in field hockey have high chances of getting ankle and overuse injuries. This study suggests the need for a guideline of injury prevention strategies that can guide coaches, sports scientists, and field hockey players. In addition, more studies are needed to focus on other pertinent information of injury as it could be the basis for prevention measures to reduce the prevalence and incidence of injury among hockey players.

## References

[1] Theilen TM, Mueller-Eising W, Bettink PW, Rolle U (2016). Injury data of major international field hockey tournaments.. Br J Sports Med..

[2] Dick R, Hootman JM, Agel J, Vela L, Marshall SW, Messina R (2007). Descriptive epidemiology of collegiate women's field hockey injuries: National Collegiate Athletic Association Injury Surveillance System, 1988-1989 through 2002-2003.. J Athl Train..

[3] Junge A, Langevoort G, Pipe A, Peytavin A, Wong F, Mountjoy M (2006). Injuries in team sport tournaments during the 2004 Olympic games.. Am J Sports Med..

[4] Orooj M, Nuhmani S, Muaidi Q (2016). Common injuries in field hockey.. Saudi J Sport Med..

[5] Ellapen TJ, Bowyer K, Van Heerden HJ (2014). Common acute and chronic musculoskeletal injuries among female adolescent field hockey players in KwaZulu-Natal, South Africa. S Afr J Sports Med..

[6] Murtaugh K (2001). Injury patterns among female field hockey players. Med Sci Sports Exerc..

[7] Murtaugh K (2009). Field hockey injuries.. Curr Sports Med Rep..

[8] Luthje P, Nurmi I, Kataja M, Belt E, Helenius P, Kaukonen JP (1996). Epidemiology and traumatology of injuries in elite soccer: a prospective study in Finland.. Scand J Med Sci Sports.

[9] Moreno-Alcaraz VJ, Cejudo A, de Baranda PB (2020). Injury types and frequency in Spanish inline hockey players. Phys Ther Sport..

[10] Scott SH, Winter DA (1991). Talocrural and talocalcaneal joint kinematics and kinetics during the stance phase of walking.. J Biomech..

[11] Nester CJ, Findlow AF, Bowker P, Bowden PD (2003). Transverse plane motion at the ankle joint.. Foot Ankle Int..

[12] Ellapen T, Abrahams S, Desai F, Narsigan S, Van Heerden HJ (2011). Prevalence of musculoskeletal pain among the South African female senior national hockey players. Postepy Rehabil..

[13] Steffen K, Andersen TE, Bahr R (2007). Risk of injury on artificial turf and natural grass in young female football players.. Br J Sports Med.

[14] Torg JS, Quedenfeld TC, Landau S (1974). The shoe-surface interface and its relationship to football knee injuries.. J Sports Med..

[15] Fischer F, Menetrey J, Herbort M, Gfoller P, Hepperger C, Fink C (2016). p 27-38.. Prevention of Injuries and Overuse in Sports..

[16] Caine D, Maffulli N, Caine C (2008). Epidemiology of injury in child and adolescent sports: injury rates, risk factors, and prevention.. Clin Sports Med..

